# Screening for diabetic retinopathy with fluorescein angiography in patients with type 1 diabetes from adolescence to adult life. A retrospective study of the past 30 years of clinical practice in a tertiary Belgian centre

**DOI:** 10.1002/edm2.304

**Published:** 2021-10-23

**Authors:** Gwendolina Conti, Laurence Postelmans, Harry Dorchy

**Affiliations:** ^1^ Department of Ophthalmology Hôpital Universitaire Brugmann Brussels Belgium; ^2^ Diabetology Clinic Hôpital Universitaire des Enfants Reine Fabiola Université Libre de Bruxelles Brussels Belgium

**Keywords:** adolescent, child, diabetic retinopathy, fluorescein angiography, type 1 diabetes

## Abstract

**Background:**

The aim of the present study was to describe the prevalence and progression of DR diagnosed by fluorescein angiography (FA) in patients with type 1 diabetes (T1D) during a 30‐year follow‐up, and the relationship with glycated haemoglobin (HbA1c).

**Materials and methods:**

We included 4325 FA reports representing 851 patients with T1D with a mean age at diagnosis of 10.4 years (range: 0.0–49.9) and followed between 1986 and 2015. Clinical characteristics of the population were collected from patients’ files. The HbA1c level was measured within a maximum period of ±1 year from the date of FA. Descriptive statistics were realized to study prevalence and progression of DR.

**Results:**

At diagnosis of incipient abnormalities, mean age was 22.8 years (range 13.7–46.9) and mean diabetes duration was 13 years (range: 4.3–29.6). Lesions requiring treatment were observed in 5.9% of the patients at a mean age of 32.4 years (range: 30.4–34.3) and a mean diabetes duration of 23.8 years (range: 19.4–28.1). On average, it took 12.9 years (range: 12.2–13.5) to progress from an incipient abnormality to a lesion requiring treatment. Mean HbA1c ± SD was 7.8 ± 1.5% over a period of 30 years.

**Conclusions:**

While it could have been expected to observe a higher prevalence of DR, our study described by far the lowest results of prevalence comparing to similar studies, probably due to a good average HbA1c over 30 years.

## Graphical abstract

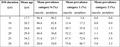

## 1 INTRODUCTION

In most western countries, diabetic retinopathy (DR) is the leading cause of blindness in the working age population.^1^ The Wisconsin Epidemiologic Study of Diabetic Retinopathy as well as the Diabetes Clinical and Complications Trial have clearly established that a better metabolic control, in term of lower glycated haemoglobin A1c (HbA1c), reduces microvascular complications.^2^, ^3^ The progression rate of microangiopathy is of major prognostic importance to every patient with type 1 diabetes (T1D) even if most of these retinal vascular abnormalities detected in adolescents and young adults with T1D do not require photocoagulation therapy.

A lot of epidemiological studies have described DR and its relationships to various risk factors.^3^, ^4^, ^5^, ^6^, ^7^, ^8^ Only a few have been realized to study the prevalence of DR by fluorescein angiography (FA).^4^

In 2018, The International Society for Pediatric and Adolescent Diabetes (ISPAD) guidelines recommended that screening for retinopathy should start from age 11 years and after 2–5 years of diabetes duration. Minimal assessment for retinopathy should be performed by ophthalmoscopy through dilated pupils by an experienced observer. The frequency of retinopathy screening should occur annually or more frequently in case of high‐risk features for visual loss.^9^

The retinal FA allows detection of early abnormalities that are undetectable by regular ophthalmoscopy. It enables the study of the vascular walls and the perturbations of the dynamic circulation. In our experience, compared with regular ophthalmoscopy, FA doubles the diagnosis of incipient retinopathy.^10^ FA allows the detection of retinal changes approximately 4 years earlier than ophthalmoscopy.^11^ A recent comparison of digital colour fundus imaging and fluorescein angiographic findings for the early detection of DR in young patients with T1D confirmed the superiority of FA.^12^

The aim of this retrospective 30‐year study was to describe the prevalence and progression of DR detected by FA in patients with T1D followed at the Diabetology Clinic of the University Children's Hospital Queen Fabiola and the Department of Ophthalmology of the Brugmann University Hospital in Brussels.

## 2 MATERIALS AND METHODS

### 2.1 Reports

**FIGURE 1:**
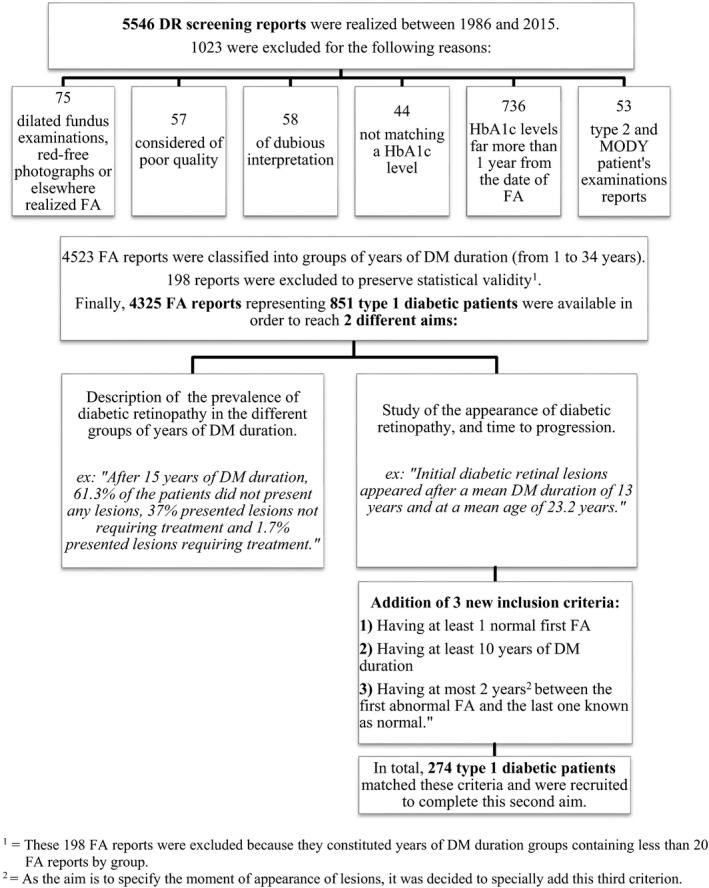
Construction of the database which is a gathering of DR screening reports from 1986 to 2015. Patient data were used differently to reach the first and the second aim of this study. ^1^These 198 FA reports were excluded because they constituted years of DM duration groups containing less than 20 FA reports by group. ^2^As the aim is to specify the moment of appearance of lesions, it was decided to specially add this third criterion

The database is a gathering of DR screening reports stored at the Diabetology Clinic of University Children's Hospital Queen Fabiola. Only screening examinations made by FA at the Department of Ophthalmology of Brugmann University Hospital matching a HbA1c level, measured within a maximum period of ±1 year from the date of FA, were recorded. In total, 5546 FA were realized between 1986 and 2015; 1023 were excluded for different reasons: 75 were dilated fundus examinations, red‐free images or FA realized elsewhere, 57 were considered of poor quality, 58 were of dubious interpretation, 44 had no corresponding HbA1c level, 6 had a corresponding HbA1c level but far more than 1 year from the date of FA, 184 were type 2 or MODY diabetic patients’ reports. From the 4523 remaining FA reports, 198 were excluded because of a lack of FA in duration groups of more than 30 years. In total, 4325 FA reports were included (Figure 1).

Through three decades, insulin treatments (but not the paediatric diabetologist, head of the team, HD), angiographic material and retina specialists protocoling FA reports, have changed.

Currently, a mix of one third phenylephrine and two thirds tropicamide is used to dilate pupils.

Detailed methodology for FA has been previously describe.^4^, ^8^, ^13^ Over time, there has been a technical evolution in angiography, from cameras using silver print (Zeiss, Canon, Topcon) to digital print acquisition as by Scanning Laser Ophthalmoscopy (SLO Heidelberg). These differences were not reported in FA protocols.

Macular and peripheral areas were described separately for each eye. While constituting the database, it has been noticed that different ophthalmologists wrote reports following different classifications which have evolved over time. Therefore, the decision was made to transfer observed lesions into three categories approved by all ophthalmologists.

Category 0 includes examinations where no retinopathy was observed.
Category 1 includes examinations where incipient retinopathy was observed. This term represents initial DR lesions such as fluorescein leakages without macular oedema, microaneurysms, microhaemorrhages, capillaries dilatations or occlusions.
Category 2 includes examinations reporting lesions requiring treatment, such as clinically significant macular oedema, retinal ischaemia and preretinal or papillary neovascularization.

All images from category 2 were reviewed in 2016 by an ophthalmologist specialized in retina. We decided to graduate those lesions according to the most commonly used DR classification, the Diabetic Retinopathy Severity Score (DRSS). It is based on seven‐field stereoscopic film‐based fundus photographs. Levels are categorized from 10 (no retinopathy) to 85 (advanced proliferative DR). Although FA is not part of the DRSS, we chose this classification because it is reproductible, validated and used in clinical trials. In Table S1, we reported a condensed version of the scale.^14^

If more than one FA was realized in the same year, only the one including the higher number of lesions was recorded. The 4422 FA reports were classified into groups of years of DM duration.

### 2.2 Subjects

In total, 4325 FA reports (a mean of 5.1 reports per patient) representing 851 patients with T1D (371 females, 480 males), followed over the past 30 years, compose the database.

### 2.3 Clinical and biological assessments

A retrospective review of these 851 patients’ files was performed. Data collected included age at diagnosis of DM, evolution of DR, patient's age, DM duration, HbA1c level and sides effects caused by intravenous injection of sodium fluorescein. All data related to age and duration were expressed in years.

The HbA1c level was measured within a maximum period of ±1 year from the date of FA with different methodologies between 1986 and 2015. The current technique is the high‐performance liquid chromatography system (HA‐8160), aligned with Diabetes Clinical and Complications Trial. In order to compare our results, each HbA1c level was first expressed in a percentage according to the actual upper normal limit (6.2%).

### 2.4 Prevalence of diabetic retinopathy

The first aim of this study was to describe the prevalence of abnormalities (categories: 0, 1 and 2, graded according to the DRSS Classification), in macular and peripheral areas, throughout years of DM duration. The different required ophthalmic treatments were described, as well as side effects due to fluorescein injection. In order to complete this first aim, data of the 851 patients were used.

### 2.5 Progression of diabetic retinopathy

The second aim of this work was to describe the progression of initial abnormalities (categories 1 and 2) and time of progression. To study this, three new inclusion criteria were added: having at least one normal FA at the beginning of the screening; having at least 10 years of DM duration; having at most a 2‐year period between the first abnormal FA and the last one known as normal. Among the 851 patients, 274 (32%) matched these criteria and were recorded.

### 2.6 Data analysis

The statistical analysis was performed using IBM SPSS® Statistics 23. Descriptive statistics were calculated for baseline characteristics of the subjects (mean, SD, median, minimum, and maximum), prevalence of retinopathy for each year of DM duration group and progression of diabetic lesions.

## 3 RESULTS

A total of 851 patients with T1D underwent 4325 DR screening examinations by FA between 1986 and 2015. Baseline characteristics of these subjects appear in Table 1. Age at DM diagnosis was 10.4 ± 6.6 years (range: 0–49.9). DM duration at the end of the study period was 18.4 ± 9.7 years (range: 2.3–51.3). Over the study period, mean HbA1c level was 7.8 ± 1.5% and in most cases >6.5 and ≤8% (53.7%).

**TABLE 1:** Baseline characteristics of the study population

Characteristics	*n* = 851
Gender (%)	
Female	371 (43.6)
Male	480 (56.4)
Age at DM onset (years)	
Mean ± SD	10.4 ± 6.58
Median (min, max)	9.8 (0.0, 49.9)
≤5	152 (17.9%)
>5–<10	292 (34.3%)
≥10	407 (47.8%)
DM duration at the end of study period (years)	
Mean ± SD	18.4 ± 9.68
Median (min, max)	16.8 (2.3, 51.3)
≤5	28 (3.3%)
>5–<10	153 (18%)
≥10	670 (78.7%)
Age at the end of study period (years)	
Mean ± SD	28.8 ± 10.37
Median (min, max)	26.3 (13.1, 70.9)
≤5	18 (2.1%)
>5–<10	362 (42.6%)
≥10	471 (55.3%)
Average HbA1c during study period (%)	
Mean ± SD	7.8 ± 1.5
Median (min, max)	7.7 (4, 18.7)
≤6.5	813 (18.4)
>6.5–≤7.5	1319 (29.8)
>7.5–≤8	1056 (23.9)
>8–≤10	941 (21.2)
>10	293 (6.6)

### 3.1 Prevalence of diabetic retinopathy

Table 2 shows the mean prevalence of retinal lesions in macular and peripheral areas according to year of DM duration.

**TABLE 2:** Description of the mean prevalence of diabetic retinopathy found in macular and peripheral areas after 5, 10, 15, 20, 25 and 30 years of DM duration. Category 0 includes examinations where no retinopathy was observed. Category 1 includes examinations where incipient retinopathy was observed. Category 2 includes examinations reporting lesions requiring treatment

DM duration (years)	Mean age (years)	Mean prevalence category 0 (%)		Mean prevalence category 1 (%)		Mean prevalence category 2 (%)	
		Macula	Periphery	Macula	Periphery	Macula	Periphery
5	17.7	96.4	96.2	3.6	3.8	0.0	0.0
10	20.2	86.6	82.8	13.4	17.2	0.0	0.0
15	24.6	68.1	62.4	30.7	35.8	1.2	1.7
20	29.4	46.8	36,0	52.2	60.2	1.1	3.8
25	34.2	31.6	23.7	67.5	72.8	0.9	3.5
30	39.5	20.0	10.0	75.0	86.7	5.0	3.3

After 10 years of DM, 13.4% and 17.2% of patients were found with incipient DR (category 1) respectively in macular and peripheral areas versus 52.2% and 60.2% after 20 years, versus 75% and 86.7% after 30 years.

Lesions requiring treatment (category 2) were observed after 15 years respectively in macular and peripheral areas in 1.2% and 1.7% of patients versus 5% and 3.5% after 30 years of DM duration.

Among 851 patients, 36 (4.2%) required ocular treatment. The decision to treat was made by retinal specialists. At the time of the first treatment, nine patients had clinically nonsignificant macular oedema, 13 patients had clinically significant macular oedema, 2 had mild non proliferative DR (grade 35), 6 had moderate non proliferative DR (grade 43–47), 14 had severe non proliferative DR (grade 53) and 9 had mild proliferative DR (grade 61).^15^

The treatment mainly consisted in photocoagulation therapy, sometimes associated with non‐steroidal anti‐inflammatory eye drops, acetazolamide tablets or anti‐vascular endothelial growth factor intravitreal injection. Two patients required vitrectomy.

The mean HbA1c, as well as the number of FA reports according to DM duration can be found in Table S2.

Considering side effects, 17 patients among 4424 (0.4%) had nauseas. In addition, one of them had reactive urticaria and another one had Quincke's oedema. No death occurred.

### 3.2 Progression of diabetic retinopathy

Data from 274 patients with T1D were used to achieve the second aim.

Considering a maximum period of 1.5 years between the first abnormal FA and the last one known as normal, age at diagnosis of category 1 lesions was 22.8 ± 6 years (range: 13.7–46.9), after a duration of 13 ± 4.7 years (range: 4.3–29.6).

The age at diagnosis of category 2 lesions was 32.4 ± 2.8 years (range: 30.4–34.3), after a duration of 23.8 ± 6.1 years (range: 19.4–28.1).

On average, it took 12.9 ± 1 years (range 12.2–13.5) to move from an incipient retinal lesion to a lesion requiring treatment (Table 3).

**TABLE 3:** Progression of DR considering mean DM duration, mean age of appearance of categories 1 and 2 lesions. Category 1 includes examinations where incipient retinopathy was observed. Category 2 includes examinations reporting lesions requiring treatment

Time from the most recent FA	Maximum of 1.5 years
Total no. of patients	274
Patients’ category 1	133 (48.5%)
Average age (years)	
Mean ± SD	22.8 ± 6
Median (min–max)	22 (13.7–46.9)
Average DM duration (years)	
Mean ± SD	13 ± 4.7
Median (min–max)	12.1 (4.3–29.6)
Patients’ category 2	2 (0.7%)
Average age (years)	
Mean ± SD	32.4 ± 2.8
Median (min–max)	32.4 (30.4–34.3)
Average DM duration (years)	
Mean ± SD	23.8 ± 6.1
Median (min–max)	23.75 (19.4–28.1)
Time between categories 1 and 2 (years)	
Mean ± SD	12.9 ± 1
Median (min–max)	12.9 (12.2–13.5)

Abbreviation: DR, diabetic retinopathy.

## 4 DISCUSSION

This study provides a unique overview of long‐term clinical practice. The main feature of this work is the description of prevalence and progression of DR diagnosed by a sensitive method, FA,^8^ in a large young population, during a very long follow‐up with available data. Our aim was to use the massive available database to publish a report looking back over the last 30 years, taking stock of what has been achieved in terms of screening.

However, this work has limitations due to a lack of data on risk factors such as blood pressure, body mass index, lipids levels, smoking, puberty and maternity. Also, DR is the only studied complication.

### 4.1 Prevalence of diabetic retinopathy

Reviewing literature, it is complicated to compare our results with other studies describing prevalence of DR in young patients with T1D. Firstly, because they mainly used fundus photographs as screening technique, secondarily because metabolic control reflected by HbA1c level and methodology differed from one study to another.

Still, considering this, we concentrated on studies using FA. After 10 years of DM, Burger et al.,^11^ in the longitudinal Berlin study, observed than 60% of patients developed retinal changes versus only 17% of our patients. After 15 years, the proportions were 90% versus 37.5%. In a study published in 2015, including 370 children aged less than 18 years with type 1 or type 2 diabetes over a 4‐year period, Geloneck et al.,^16^ using simple dilated eye examination, found no cases of DR. However mean HbA1c was high, 8.6% (range: 5–14), but mean diabetes duration was low, 5.2 years (range: 0.1–16.2). Based on these results obtained with a less sensitive method, they suggested that screening for DR should start at 15 or 5 years after DM onset but, thanks to FA, we described earlier lesions starting at 13 years of age and after 4 years of DM.

The relatively low prevalence of DR in our study could be due to the degree of glycaemic control (HbA1c 7.8 ± 1.5% (range: 4–18.7)).

Objectively, our study shows that moderate and severe side effects of FA are very rare in youth.

### 4.2 Progression of diabetic retinopathy

This study described the apparition of the first signs of DR at 13 years of age and after 4 years of DM, approximately at the same period 2018 ISPAD guidelines recommended to start screening.^9^

Our results were consistent with a study we published more than 25 years ago.^17^

From our point of view, the practical interest of an early diagnosis of DR is to motivate both the patient and the multidisciplinary paediatric diabetology team to obtain the best possible metabolic control to avoid serious and irreversible complications. The HbA1c targets, expectations and goals that diabetes care professionals have for their patients is determinant in metabolic outcomes.^18^ Considering the ophthalmological point of view, the optimal period for therapy is often missed when visual impairment occurs. Therefore, early recognition, prevention and treatment of DR should be of major consideration.^19^

### 4.3 Recent breakthroughs in screening

New imaging systems could be useful in screening, in grading, in the treatment and the follow‐up of DR. While enhancement of smartphone‐based retinal imaging holds great promise to increase the accessibility of retinal screening,^20^ it seems to be more useful in DR lesions follow‐up then in early diagnosis in children. It is based on wide field mydriatic retinal photographs which has been demonstrated less sensitive than FA in revealing information about retinal vascular pathology after a short disease time.^13^ Also, this article showed that identifying minor DR signs at a stage with unaffected visual acuity constitutes an alert for a stricter glycaemic control and more frequent fundus examination.

Optical coherence tomography angiography could potentially apply. It visualizes vascular changes including intraretinal abnormalities and neovascularization, measuring foveal avascular zone, superficial and deep vessel density. These might be sensitive imaging biomarkers to define early DR. This method is rapid and noninvasive. It uses motion of erythrocytes to detect the flow in the retinal capillaries. It has the advantage of visualizing the different layers of FAZ separately^21^ but it is not yet used in screening for DR because of the limited field and a relative sensitivity to detect microaneurysms.

## 5 CONCLUSION

While it could have been expected to observe a higher prevalence of DR because FA allows the detection earlier than fundus photographs, our study described by far the lowest results of DR prevalence comparing to similar studies. These good results are probably due to a good average HbA1c^22^ over a period of 30 years but the impact of other risk factors was not considered in this study. Other techniques are being studied to propose another way of screening. Anyway, most important aspect should always be the benefit of patients with T1D in order to prevent later definitive complications.

[Correction added on 18 December 2021 after first online publication: Citation to reference Dorchy (2015) has been added and reference list updated.]

## CONFLICT OF INTEREST

The authors declare that the research was conducted in the absence of any commercial or financial relationships that could be construed as a potential conflict of interest.

## AUTHOR CONTRIBUTIONS

Gwendolina Conti: Conceptualization (lead); Data curation (lead); Formal analysis (lead); Investigation (lead); Methodology (lead); Project administration (equal); Writing‐original draft (lead); Writing‐review & editing (lead). Laurence Postelmans: Conceptualization (equal); Data curation (equal); Methodology (equal); Project administration (equal); Resources (supporting); Supervision (supporting); Validation (supporting); Writing‐original draft (supporting); Writing‐review & editing (supporting). Harry Dorchy: Conceptualization (equal); Data curation (equal); Formal analysis (equal); Investigation (equal); Methodology (equal); Project administration (equal); Resources (lead); Supervision (lead); Validation (lead); Writing‐original draft (supporting); Writing‐review & editing (supporting).

## ETHICS APPROVAL STATEMENT

This is a retrospective study without new intervention—whether physical, psychological, or chemical—concerning the routine follow‐up of young patients with diabetes.

## PERMISSION TO REPRODUCE MATERIAL FROM OTHER SOURCES

None to declare. We don't reproduce any material from other sources.

## Supporting information

Table S1‐S2

## Data Availability

All data are personal and come from the patients’ medical reports.
