# Supplementary material for: Screening for diabetic retinopathy with fluorescein angiography in patients with type 1 diabetes from adolescence to adult life. A retrospective study of the past 30 years of clinical practice in a tertiary Belgian centre
Source: Endocrinol Diabetes Metab. 2021 Oct 23;5(1):e00304. doi: 10.1002/edm2.304 (PMC8754238; doi:10.1002/edm2.304)
Supplement: Supplementary file 1 — Table S1‐S2 [file EDM2-5-e00304-s001.docx]

**Supplementary table 1.** The scale used to graduate diabetic retinopathy lesions requiring treatment is a condensed version of the Diabetic Retinopathy Severity Score.^14,15^

| **Level** | **Severity** | **Definition** |
| --- | --- | --- |
| 10 | No retinopathy | Diabetic retinopathy absent |
| 20 | Very mild nonproliferative diabetic retinopathy | Microaneurysms only |
| 35 | Mild nonproliferative diabetic retinopathy | Microaneurysms plus hard exudates, soft exudates and/or mild retinal hemorrhages. |
| 43 | Moderate nonproliferative diabetic retinopathy | Microaneurysms plus mild intraretinal microvascular abnormalities or moderate retinal hemorrhages. |
| 47 | Moderate nonproliferative diabetic retinopathy | More extensive intraretinal microvascular abnormalities, severe retinal hemorrhages, or venous beading in one quadrant only. |
| 53 | Severe nonproliferative diabetic retinopathy | Severe retinal hemorrhages in four quadrants, or venous beading in at least two quadrants, or moderately severe intraretinal microvascular abnormalities in at least one quadrant. |
| 61 | Mild proliferative diabetic retinopathy | New vessels elsewhere <½-disc area in one or more quadrants, or greater including new vessels on or within 1-disc diameter of optic nerve and/or vitreous hemorrhage. |

**Supplementary table 2.** Description of the prevalence of lesions (categories 0, 1, 2) in different years of DM duration

| **DM duration (y)** | **1** | **2** | **3** | **4** | **5** | **6** | **7** | **8** | **9** | **10** | **11** | **12** | **13** | **14** | **15** |
| --- | --- | --- | --- | --- | --- | --- | --- | --- | --- | --- | --- | --- | --- | --- | --- |
| Number of FA | 20 | 64 | 216 | 257 | 275 | 269 | 263 | 268 | 262 | 258 | 248 | 187 | 212 | 186 | 173 |
| **HbA1c** |  |  |  |  |  |  |  |  |  |  |  |  |  |  |  |
| Mean | 8,0 | 8,0 | 8,0 | 8,0 | 8,1 | 8,1 | 8,1 | 8,0 | 8,0 | 8,2 | 8,1 | 8,2 | 8,2 | 8,1 | 8,1 |
| ± SD | 0,4 | 0,2 | 0,1 | 0,1 | 0,1 | 0,1 | 0,1 | 0,1 | 0,1 | 0,1 | 0,1 | 0,1 | 0,1 | 0,1 | 0,1 |
| Min | 5,6 | 5,8 | 4,4 | 5,4 | 4,2 | 4,8 | 4,5 | 5,2 | 5,4 | 5,7 | 4,2 | 4,9 | 4,9 | 4,9 | 5,4 |
| Max | 13,4 | 13,8 | 14,9 | 14,1 | 17,0 | 15,4 | 18,7 | 14,2 | 15,9 | 14,0 | 15,0 | 17,9 | 17,8 | 15,4 | 15,1 |
| **Macula OD** |  |  |  |  |  |  |  |  |  |  |  |  |  |  |  |
| Category 0 (%) | 100 | 98,4 | 97,7 | 96,5 | 97,1 | 93,7 | 91,6 | 90 | 90,5 | 87,6 | 79 | 77,5 | 75,0 | 69,7 | 68,6 |
| Category 1 (%) | 0 | 1,6 | 2,3 | 3,1 | 2,9 | 6,3 | 8,4 | 10 | 9,5 | 12,4 | 20,6 | 22,5 | 24,5 | 29,7 | 30,2 |
| Category 2 (%) | 0 | 0 | 0 | 0 | 0 | 0 | 0 | 0 | 0 | 0 | 0,4 | 0 | 0,5 | 0,5 | 1,2 |
| **Peiphery OD** |  |  |  |  |  |  |  |  |  |  |  |  |  |  |  |
| Category 0 (%) | 100 | 95,3 | 97,7 | 97,7 | 96,7 | 94,8 | 92,4 | 89,6 | 88,6 | 82,2 | 77,8 | 71,7 | 74,1 | 68,6 | 63,6 |
| Category 1 (%) | 0,0 | 4,7 | 2,3 | 2,3 | 3,3 | 5,2 | 7,6 | 10,4 | 11,4 | 17,8 | 22,2 | 26,7 | 25,5 | 29,7 | 34,7 |
| Category 2 (%) | 0 | 0 | 0 | 0 | 0 | 0 | 0 | 0 | 0 | 0 | 0,4 | 1,6 | 0,5 | 1,6 | 1,7 |
| **Macula OS** |  |  |  |  |  |  |  |  |  |  |  |  |  |  |  |
| Category 0 (%) | 100 | 98,4 | 97,2 | 97,3 | 95,7 | 93,0 | 93,9 | 88,8 | 91,3 | 85,7 | 79,8 | 74,9 | 75,9 | 66,5 | 67,6 |
| Category 1 (%) | 0 | 1,6 | 2,8 | 2,3 | 4,3 | 7 | 6,1 | 11,2 | 8,7 | 14,3 | 19,8 | 25,1 | 23,6 | 33 | 31,2 |
| Category 2 (%) | 0 | 0 | 0 | 0 | 0 | 0 | 0 | 0 | 0 | 0 | 0,4 | 0 | 0,5 | 0,5 | 1,2 |
| **Periphery OS** |  |  |  |  |  |  |  |  |  |  |  |  |  |  |  |
| Category 0 (%) | 100 | 95,3 | 98,2 | 96,9 | 95,7 | 95,6 | 92,8 | 89,6 | 89,4 | 83,3 | 79 | 71,1 | 74,1 | 67,6 | 61,3 |
| Category 1 (%) | 0 | 4,7 | 1,8 | 3,1 | 4,3 | 4,4 | 7,2 | 9,7 | 10,6 | 16,7 | 21 | 27,8 | 25,5 | 30,8 | 37,0 |
| Category 2 (%) | 0 | 0 | 0 | 0 | 0 | 0 | 0 | 0 | 0 | 0 | 0 | 1,1 | 0,5 | 1,6 | 1,7 |
| **DM duration (y)** | **16** | **17** | **18** | **19** | **20** | **21** | **22** | **23** | **24** | **25** | **26** | **27** | **28** | **29** | **30** |
| Number of FA | 142 | 141 | 147 | 105 | 94 | 83 | 71 | 79 | 59 | 57 | 50 | 42 | 30 | 37 | 30 |
| **HbA1c** |  |  |  |  |  |  |  |  |  |  |  |  |  |  |  |
| Mean | 8,0 | 8,0 | 7,9 | 7,8 | 8,0 | 8,0 | 7,8 | 7,9 | 7,7 | 7,7 | 7,7 | 7,7 | 8,0 | 7,6 | 7,7 |
| ± SD | 0,1 | 0,1 | 0,1 | 0,1 | 0,1 | 0,1 | 0,2 | 0,2 | 0,1 | 0,1 | 0,2 | 0,2 | 0,3 | 0,2 | 0,2 |
| Min | 5,6 | 4,7 | 5,6 | 4,7 | 5,5 | 5,2 | 5,6 | 5,6 | 5,7 | 5,8 | 5,3 | 5,3 | 5,8 | 5,8 | 5,5 |
| Max | 16,0 | 13,9 | 12,9 | 12,5 | 12,1 | 11,2 | 12,1 | 11,9 | 11,7 | 12,0 | 11,2 | 10,4 | 10,7 | 9,6 | 10,0 |
| **Macula OD** |  |  |  |  |  |  |  |  |  |  |  |  |  |  |  |
| Category 0 (%) | 64,3 | 58,9 | 59,2 | 55,8 | 47,3 | 50 | 36,6 | 41,8 | 30,5 | 29,8 | 32,7 | 31 | 36,7 | 27,8 | 23,3 |
| Category 1 (%) | 34,3 | 39 | 37,4 | 42,3 | 51,6 | 48,8 | 63,4 | 55,7 | 67,8 | 68,4 | 67,3 | 66,7 | 60 | 75 | 73,3 |
| Category 2 (%) | 1,4 | 2,1 | 3,4 | 1,9 | 1,1 | 1,2 | 0 | 2,5 | 1,7 | 1,8 | 0,0 | 2,4 | 3,3 | 2,8 | 3,3 |
| **Peiphery OD** |  |  |  |  |  |  |  |  |  |  |  |  |  |  |  |
| Category 0 (%) | 51,7 | 47,5 | 46,3 | 35,6 | 36,6 | 31,7 | 26,8 | 24,1 | 27,1 | 24,6 | 30,6 | 19 | 26,7 | 11,1 | 10 |
| Category 1 (%) | 47,6 | 51,8 | 52,4 | 61,5 | 60,2 | 64,6 | 71,8 | 69,6 | 69,5 | 71,9 | 67,3 | 73,8 | 66,7 | 88,9 | 83,3 |
| Category 2 (%) | 0,7 | 0,7 | 1,4 | 2,9 | 3,2 | 3,7 | 1,4 | 6,3 | 3,4 | 3,5 | 2 | 7,1 | 6,7 | 5,6 | 6,7 |
| **Macula OS** |  |  |  |  |  |  |  |  |  |  |  |  |  |  |  |
| Category 0 (%) | 60,7 | 58,9 | 57,1 | 51 | 46,2 | 47,6 | 40,8 | 43,0 | 30,5 | 33,3 | 26,5 | 28,6 | 33,3 | 25,0 | 16,7 |
| Category 1 (%) | 38,6 | 39,0 | 41,5 | 47,1 | 52,7 | 50 | 59,2 | 54,4 | 64,4 | 66,7 | 73,5 | 66,7 | 63,3 | 77,8 | 76,7 |
| Category 2 (%) | 0,7 | 2,1 | 1,4 | 1,9 | 1,1 | 2,4 | 0 | 2,5 | 5,1 | 0 | 0 | 4,8 | 3,3 | 2,8 | 6,7 |
| **Periphery OS** |  |  |  |  |  |  |  |  |  |  |  |  |  |  |  |
| Category 0 (%) | 54,5 | 49,6 | 44,9 | 32,7 | 35,5 | 35,4 | 29,6 | 24,1 | 20,3 | 22,8 | 24,5 | 16,7 | 26,7 | 11,1 | 10 |
| Category 1 (%) | 44,8 | 49,6 | 54,4 | 63,5 | 60,2 | 61,0 | 67,6 | 68,4 | 72,9 | 73,7 | 71,4 | 76,2 | 66,7 | 91,7 | 90 |
| Category 2 (%) | 0,7 | 0,7 | 0,7 | 3,8 | 4,3 | 3,7 | 2,8 | 7,6 | 6,8 | 3,5 | 0 | 7,1 | 6,7 | 0 | 0 |
